# Takayasu arteritis a cause of hypertensive disorder of pregnancy: a case report

**DOI:** 10.1186/s13256-017-1534-6

**Published:** 2018-01-17

**Authors:** Jesus Lumbreras-Marquez, Roberto Arturo Castillo-Reyther, Salvador De-la-Maza-Labastida, Fernando Vazquez-Alaniz

**Affiliations:** 1 Gynaecology & Obstetric Department. Hospital Central “Dr. Ignacio Morones Prieto”. Health Ministry of San Luis Potosi, Avenida Venustiano Carranza #2395, Zona Universitaria, 78290 San Luis, SLP Mexico; 2 Clinical laboratory department, Hospital General 450. Health Ministry of Durango, Blvd. Jose Maria Patoni #403, Col. El Cipres, 34206 Durango, DGO Mexico

**Keywords:** Refractory hypertension, Takayasu arteritis, Hypertensive disorder in pregnancy

## Abstract

**Background:**

Takayasu arteritis is a rare, chronic, granulomatous systemic vasculitis of unknown etiology and a few cases have been reported in pregnancy. In pregnancies concomitant with Takayasu arteritis or after diagnosis, Takayasu arteritis negatively affects pregnancy by increasing 13-fold the odds of complications such as hypertensive disorders. The best recommendations in this scenario are still to be made.

**Case presentation:**

We present a case of 21-year-old, gravid 1, Mexican woman of Mestizo descent with chronic hypertension diagnosed since she was 15-years old who presented severe hypertension during pregnancy (early second trimester); the diagnosis of hypertensive disorder of pregnancy was ruled out requiring first-line and second-line antihypertensive therapy without serious associated maternal or fetal morbidity.

**Conclusions:**

Takayasu arteritis and pregnancy play an important role in maternal and fetal outcomes. Efforts should be made to further investigate the Takayasu arteritis diagnosis in pregnant women with refractory hypertension.

## Background

Takayasu arteritis (TA) is a primary systemic vasculitis of unknown etiology that leads to stenotic and occlusion changes [1]. It was first described in a Japanese woman in 1908 by the ophthalmologist Mikito Takayasu who reported a case of a 21-year-old woman with the association of retinal arteriovenous anastomosis, syncope, and pulseless superior extremities [2]. It affects women predominantly (ratio of 4:1, female to male) [3]. The pathophysiology of TA has been described as a chronic inflammatory disease affecting the aorta and its branches in a progressive pattern, which can lead to secondary hypertension, retinopathy, cardiac pathology, stroke, and death at an early age [4]. Histopathologic inspection shows that mononuclear infiltration of the adventitia occurs early in the disease [5]. As the inflammatory process continues, a panarteritis occurs, fibrosis of the media and thickening of the intima lead to compromise of vessel lumens, resulting in vessel stenosis. No single laboratory test is available to confirm the diagnosis [6]. The diagnosis is based on the combination of clinical history, physical examination, clinical suspicion, and vascular imaging techniques. The etiology is unknown and treatment is aimed at controlling the inflammatory process and preventing secondary sequelae, particularly systemic arterial hypertension [7].

In pregnant women, there are few cases reported. However, when it is present it can emerge as a hypertensive disorder of pregnancy (HDP). HDP is the most common medical complication, affecting approximately 5 to 10% of all pregnancies. Despite advances in obstetric medicine, HDP remains the second highest cause of maternal mortality worldwide [8]. By convention, the threshold for diagnosis of hypertension in pregnancy is blood pressure (BP) levels of ≥ 140 mmHg systolic and/or ≥ 90 mmHg diastolic, confirmed by two readings at rest 4 to 6 hours apart. There is a large amount of evidence that preeclampsia (PE) may occur in women with chronic hypertension and that the prognosis for mother and fetus is much worse than with either condition alone [9]. On the other hand, in pregnancies concomitant with TA or after diagnosis, TA negatively affects pregnancy by increasing 13-fold the odds of complications such as hypertensive disorders (mainly due to arterial hypertension) and TA activity is independently associated with poor pregnancy outcomes. Obstetric complications are more common in women who are pregnant and diagnosed with TA than in those pregnant women for whom the diagnosis of TA was made before pregnancy [10].

## Case presentation

**Fig. 1:**
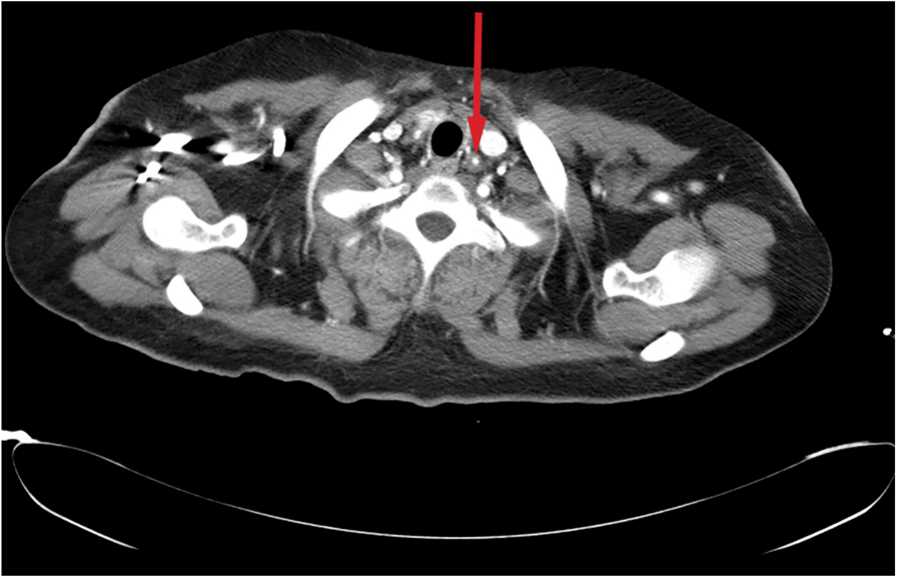
Chest angiography/mediastinal window shows a concentric wall thickening of the left subclavian artery (red arrow)

**Fig. 2:**
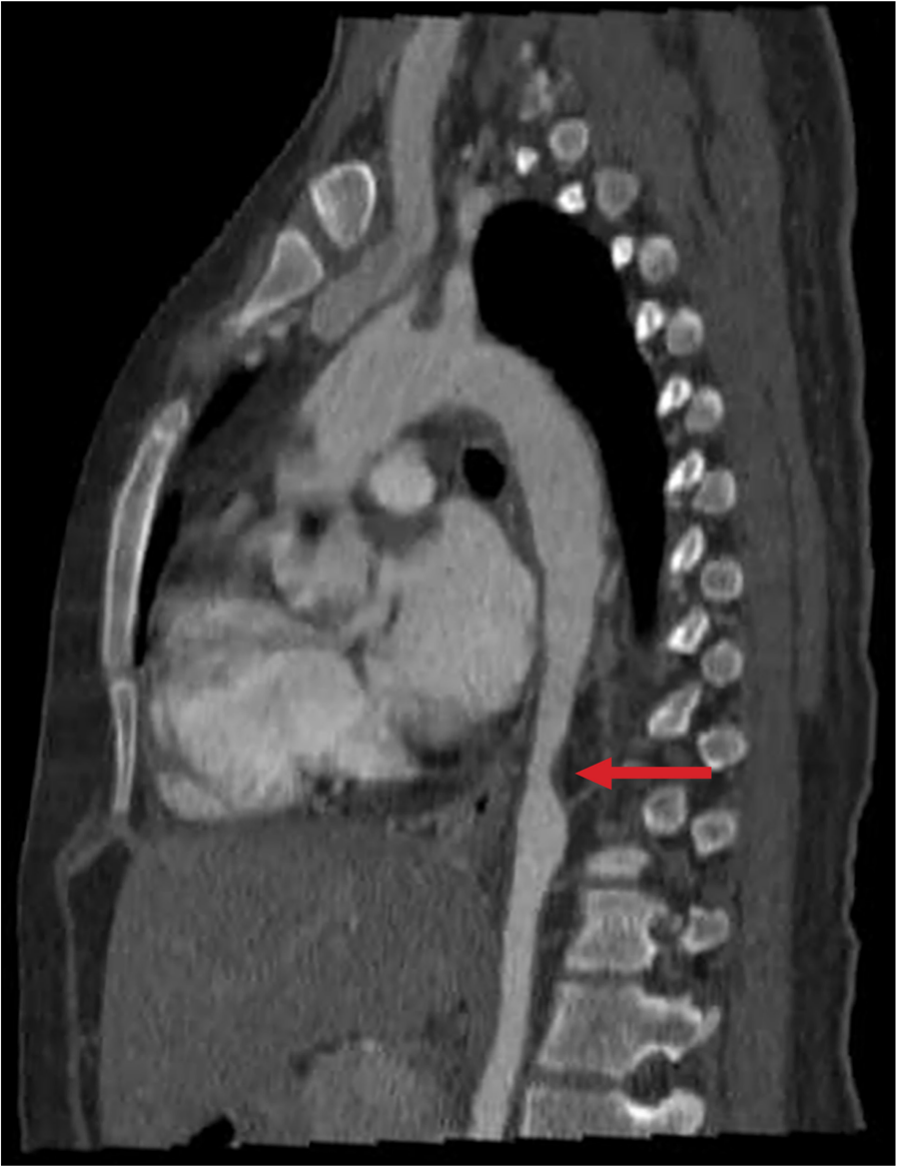
Sagittal reformatted angiotomography shows the descending aorta with an irregular thickening and stenosis of the lumen (red arrow)

A 21-year-old, gravid 1 singleton, Mexican woman of Mestizo descent, living in a rural zone, known to have chronic hypertension that was diagnosed when she was 15-years old managed prior to pregnancy with enalapril, which is an angiotensin-converting enzyme (ACE) inhibitor (dose 10 mg/12 hours), with apparently good BP control. We met her for the first time in our emergency room in January 2015; she was referred by a general physician of the Central Hospital “Dr Ignacio Morones Prieto”, with an arterial BP of 180/100 mmHg and ongoing pregnancy of 17 5/7 weeks of gestation. At first evaluation, she was found only with mild headache and without any other signs or symptoms of any other organic failure. During a physical examination, she presented a holosystolic murmur in the pulmonary area and a structurally normal heart (assessed by transthoracic echocardiogram). During further evaluation, she presented a protein/creatinine ratio (P/C ratio) of 0.09 mg/dL, a 24-hour urine protein test of 100 mg/24 hours, and the rest of the laboratory results (liver, renal, and hematological components) were within normal parameters. She was managed with methyl dopamine and nifedipine (500 mg/6 hours and 10 mg/6 hours respectively) with good response and discharged to external consultation. In the follow up, she had a second hospitalization at 27 weeks of gestation with uncontrolled BP in which PE was ruled out (24-hour urine protein test of 100 mg/24 hours). A few weeks later, she was admitted to hospital with a gestational age of 33 weeks due to severe hypertension: 190/100 mmHg with a P/C ratio of 0.17 mg/dL, a 24-hour protein in urine collection without any signs or symptoms of organ damage, and with an adequate fetal growth percentile. After an aggressive management requiring sodium nitroprusside (dose 1 μg/kg per minute) and a refractory hypertension reaching systolic BP of 220 mmHg it was decided to opt for therapeutic interruption of pregnancy given the risk of adverse maternal and fetal outcomes. After corticosteroid scheme, a live neonate of 33 5/7 weeks, weight of 2190 g, and Apgar score of 9 at 5 minutes was obtained by cesarean section without requiring Neonatal Intensive Care Unit admission. In postpartum period our patient required six antihypertensive drugs (minoxidil, carvedilol, methyl dopamine, amlodipine, enalapril, and prazosin). Assessment with magnetic resonance angiography (MRA) found a transmural thickening of the supra-aortic vessels showing a concentric wall thickening of her left subclavian artery, her brachiocephalic artery, her right common carotid artery, and her left common carotid artery (Fig. 1), her descending aorta showed an irregular thickening and stenosis of the lumen (Fig. 2), establishing a diagnosis of TA. After puerperium, she was managed with rituximab and followed by the Rheumatology service of the Central Hospital “Dr Ignacio Morones Prieto”.

## Discussion

First described in Japanese women, TA is recognized as a chronic inflammatory vasculitis of unknown origin affecting large vessels, predominantly the aorta and its main branches [10]. Although TA has been considered a rare disease, it is more common in Asian women in comparison to white women; however, recent surveys prove that the disease can be seen in all ethnicities around the world [11]. Since 1990, The American College of Rheumatology has proposed the diagnostic criteria for the disease (Table 1) [12]. It is important to consider that TA can affect women during their reproductive age because the majority of the cases develop an asymptomatic disease, making the diagnosis very difficult as in our case. It is also important to consider the high cardiovascular risk coexisting in pregnancy, unknown until a diagnosis is established. There have been cases reported of adverse cardiovascular events in pregnant women with TA [4].

**Table 1:** Clinical diagnosis of Takayasu arteritis criteria^§^

Clinical diagnosis of TA criteria	
Age at disease onset ≤40 year Claudication of extremities Decreased brachial artery pulse Systolic blood pressure difference >10 mm Hg between 2 arms Bruit over subclavian arteries or aorta Arteriographic evidence of narrowing or occlusion of aorta, its primary branches or large arteries in the proximal upper or lower extremities.	
Diagnosis TA requires at least 3 of the 6 criteria.	

^§^The American College of Rheumatology

Furthermore, HDP is a major concern during pregnancy given the risk of adverse maternal and perinatal outcomes. The etiology of HDP remains unknown, despite decades of extensive research principally focused on the role of the placenta [13]. Pregnancies complicated with TA carry a number of considerable complications such as sustained refractory hypertension, superimposed PE, congestive heart failure, and progression of renal involvement as well as an increased likelihood of low birth weight neonates [14]. In our case, the two main problems were presented during pregnancy: severe refractory hypertension and occlusive vascular affection. Management and therapy are a real challenge, as seen in our case, which makes a multidisciplinary approach necessary and the use of several antihypertensive drugs, according to American College of Obstetricians and Gynecologists recommendations [15]. In addition, the concomitant presentation of intrauterine growth restriction (IUGR) and HDP lead to complex decision making. An increase in rate of maternal and fetal complications in post-diagnosis pregnancies compared with pre-diagnosis pregnancies (worsening of hypertension, 14% vs 3%; PE, 10% vs 0%; and prematurity, 10% vs 2%) [11] has been reported. Conventional angiography is considered the gold standard for the diagnosis; currently it is frequently replaced by computed tomography or MRA in routine practice [11]. In terms of medical treatment, a good response to corticosteroids was reported and accumulating evidence also shows that biological agents, such as anti-tumor necrosis factor (TNF) agents tocilizumab and rituximab, could be used effectively in refractory cases [11].

In this report, we describe the clinical course of a 21-year-old pregnant Mexican Mestizo woman with severe and uncontrollable hypertension. We expected to face complications related to HDP such as PE and IUGR, in agreement with the complications reported by Singh *et al.* [2] who found that in pregnancies complicated with TA, PE and IUGR have reported frequencies of 10% and 40% respectively in patients without renal artery involvement and frequencies of 20% to both complications in cases with renal artery involvement. Both these complications were ruled out in the comprehensive evaluation and management of our patient. In previous reports only a refractory hypertension case was found and it was in concordance with complications reported by Assad *et al*. [14], also, it is well known that severe hypertension can cause central nervous system injury [15]. In our case, despite aggressive medical treatment, our patient’s systolic BP levels reached 220 mmHg and we decided to medically interrupt the pregnancy by cesarean section to avoid further morbidity and mortality. After delivery and despite prematurity, which is a common complication in these cases consistent with the findings reported by Comarmond *et al*. [10], the neonatal outcome was considered favorable, with a good Apgar score and without any condition requiring admission in the Neonatal Intensive Care Unit. In addition, our patient did not develop any severe, life-threatening complication and she had a satisfactory evolution with proper evaluation and management in the postpartum period by the Rheumatology division.

## Conclusions

Our acquired experience indicates that BP levels in TA and pregnancy play an important role in maternal and fetal outcomes. Efforts should be made with a multidisciplinary collaboration to further investigate the TA diagnosis or any other differential diagnosis of HDP in pregnant women with refractory hypertension.

## Abbreviations

ACE: Angiotensin-converting enzyme
BP: Blood pressure
HDP: Hypertensive disorder of pregnancy
IUGR: Intrauterine growth restriction
MRA: Magnetic resonance angiography
P/C ratio: Protein/creatinine ratio
PE: Preeclampsia
TA: Takayasu arteritis
TNF: Anti-tumor necrosis factor
